# Regulation of Photochemical Energy Transfer Accompanied by Structural Changes in Thylakoid Membranes of Heat-Stressed Wheat

**DOI:** 10.3390/ijms151223042

**Published:** 2014-12-11

**Authors:** Yoko Marutani, Yasuo Yamauchi, Akihito Miyoshi, Kanako Inoue, Ken-ichi Ikeda, Masaharu Mizutani, Yukihiro Sugimoto

**Affiliations:** 1 Graduate School of Agricultural Science, Kobe University, 657-8501 Kobe, Japan; E-Mails: yoko.marutani@stu.kobe-u.ac.jp (Y.M.); i-kana@people.kobe-u.ac.jp (K.In.); ikeken@phoenix.kobe-u.ac.jp (K.Ik.); mizutani@gold.kobe-u.ac.jp (M.M.); yukihiro@kobe-u.ac.jp (Y.S.); 2 Faculty of Agriculture, Kobe University, 657-8501 Kobe, Japan; E-Mail: miyoshi.akihito.53v@st.kyoto-u.ac.jp

**Keywords:** heat stress, state transition, wheat, thylakoid membrane, photosystem, phosphorylation

## Abstract

Photosystems of higher plants alleviate heat-induced damage in the presence of light under moderate stressed conditions; however, in the absence of light (*i.e.*, in the dark), the same plants are damaged more easily. (Yamauchi and Kimura, 2011) We demonstrate that regulating photochemical energy transfer in heat-treated wheat at 40 °C with light contributed to heat tolerance of the photosystem. Chlorophyll fluorescence analysis using heat-stressed wheat seedlings in light showed increased non-photochemical quenching (NPQ) of chlorophyll fluorescence, which was due to thermal dissipation that was increased by state 1 to state 2 transition. Transmission electron microscopy revealed structural changes in thylakoid membranes, including unstacking of grana regions under heat stress in light. It was accompanied by the phosphorylation of thylakoid proteins such as D1 and D2 proteins and the light harvesting complex II proteins Lhcb1 and Lhcb2. These results suggest that heat stress at 40 °C in light induces state 1 to state 2 transition for the preferential excitation of photosystem I (PSI) by phosphorylating thylakoid proteins more strongly. Structural changes of thylakoid membrane also assist the remodeling of photosystems and regulation of energy distribution by transition toward state 2 probably contributes to plastoquione oxidation; thus, light-driven electrons flowing through PSI play a protective role against PSII damage under heat stress.

## 1. Introduction

High temperatures under recent climate changes is a major environmental constraint for plant production because photosynthesis, which includes photochemical reactions as well as carbon assimilation, is a very heat-sensitive process [1]. Rubisco activase is an enzyme that maintains Rubisco in an active state during the carbon assimilation process and is highly sensitive to heat denaturation. Inactivation of Rubisco activase leads to a limitation during carbon assimilation [2]. Photosystem II (PSII) photochemical reactions exhibit higher susceptibility to heat stress [3]. Heat stress affects electron flow both on the donor side and the acceptor side of PSII. The water-oxidation system of PSII is believed to be the first step to be damaged by heat stress [4]. The electron flow from Q_A_ to Q_B_ in PSII has also been reported to be damaged at high temperatures [5]. In contrast, photosystem I (PSI) is more heat tolerant than PSII [6].

Plants have multiple protective mechanisms against heat stress, including the induction of heat shock proteins to protect PSII from heat-induced damage by stabilizing protein folding and preventing protein aggregation [7,8], isoprene emissions to stabilize thylakoid membranes [9,10,11], and a reactive oxygen species scavenging system mediated by antioxidants such as ascorbic acid, tocopherols, and glutathione [12]. In addition, a change in energy transfer in photosystems is believed to alleviate heat-induced damage, e.g., thermal dissipation and state transition [10].

State transition is a well-studied phenomenon that occurs in changing light conditions in photosynthetic organisms such as higher plants, cyanobacteria and green algae. However, the light condition that induces state transition differs among species. In green algae, which have been extensively studied, it is believed to be an acclimation response that redistributes excitation energy between PSII and PSI in the short term [13]. In contrast, it has been recently demonstrated that state transition is a long-term acclimation to various natural light conditions in higher plants and that a part of the light-harvesting chlorophyll-binding protein II (LHCII) is phosphorylated and behaves as an effective PSI antenna [14]. State transition is triggered by changes in the redox state of the plastoquinone (PQ) pool. Reduction of PQ activates LHCII phosphorylation, which initiates the subsequent migration of LHCII to PSI (state 1 to state 2 transition). Thus, various environmental stressors in higher plants, including heat stress, tend to affect state transition through a change in energy distribution between the photosystems [15]. Moderate heat stress induces increased energy transfer to PSI at the expense of PSII [16] and migration of phosphorylated LHCII from grana stack to stroma lamellae [17], suggesting a state 1 to state 2 transition, with LHCIIs migrating from PSII to PSI.

In this study, we focused on the regulation of photochemical energy transfer with structural and biochemical changes in thylakoid membranes in response to heat stress using normal-growing wheat, heat-stressed wheat in the presence of light, and wheat recovering from heat stress. The results revealed that heat stress induces state 2; we observed increased non-photochemical quenching (NPQ) of chlorophyll fluorescence, LHCII phosphorylation and unstacked grana regions in thylakoid membranes in response to heat stress.

## 2. Results and Discussion

### 2.1. Increase in NPQ (Non-Photochemical Quenching) of Chlorophyll Fluorescence and Unstacked Region in Thylakoid Membranes under Heat Stress

A temperature of approximately 40 °C is perilous for many plant species [18]. Also in wheat plants, 40 °C is a potentially critical temperature for decreasing photosynthetic activity. We previously reported the mechanism for the damage caused by a rapid decrease in the maximum photochemical quantum yield of PSII (*F_v_*/*F_m_*) in seedlings within 1 h of heat treatment at 40 °C in the absence of light [19]. Our data suggest that in the dark, the enhanced introduction of reducing power from stroma into thylakoid membranes that occurs at temperatures >40 °C causes over-reduction of PQ, resulting in damage to the D1 protein. However, *F_v_*/*F_m_* did not decrease under a 40 °C heat treatment in light [19], indicating that light drives a system that protects PSII from damage caused by over-reduction of PQ at high temperatures.

**Figure 1:**
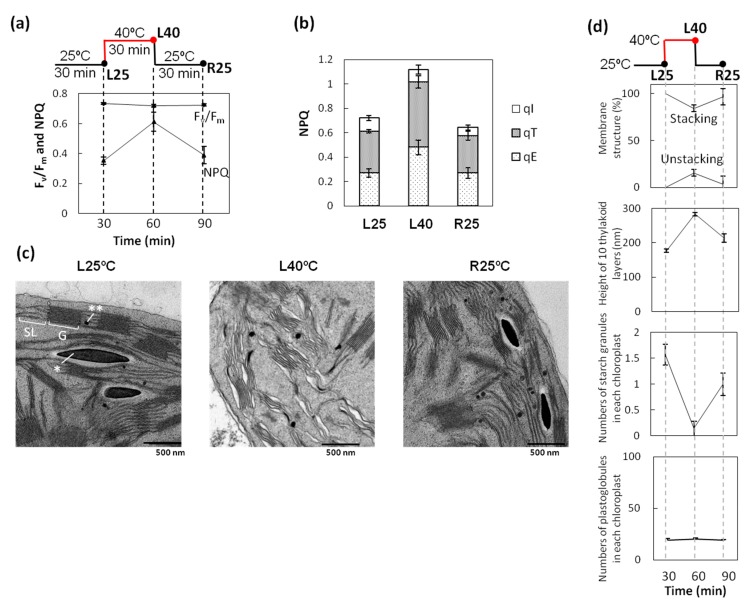
Heat treatment in light induced increase of NPQ (non-photochemical quenching) and unstacking of thylakoid membrane. (**a**) PSII (Photosystem II) maximum quantum efficiency (*F_v_*/*F_m_*). One-week-old wheat seedlings were heat-stressed under light (100 μmol photons m^−2^·s^−1^; L40). L40 wheat recovered at 25 °C was used as R25. Non-heat-stressed wheat was used as the control L25 plant. Actinic light (AL), 190 µmol photons m^−2^·s^−1^. Error bars indicate ± standard errors (SEs) (*n* = 3); (**b**) Non-photochemical quenching (NPQ), qE, qT, and qI of each sample. AL, 820 µmol photons m^−2^·s^−1^; (**c**) Electron micrographs of wheat leaves: **left** panel, control plant; **middle** panel, plant heat-stressed in light (100 µmol photons m^−2^·s^−1^); **right** panel, plant recovered in light from heat stress at 25 °C. Single and double asterisks indicate a starch granule and a plastoglobule, respectively. SL, stroma lamellae. G, Grana. Bars = 500 nm; and (**d**) Graphs: **top** panel, percentage of stacked and unstacked isolated wheat thylakoid membranes. (*n* = 3); **Upper middle** panel, height of 10 thylakoid layers (nm); **Lower middle** panel, starch granules in a chloroplast; **bottom** panel, numbers of plastoglobules in each chloroplast. Error bars indicate ± SEs (*n* = 50). Note that L40 chloroplasts altered thylakoid membranes but not those of L25 and R25.

To assess the change in energy distribution of photochemical reactions in heat-stressed wheat seedlings in the presence of light, we performed chlorophyll fluorescence analysis under different temperatures (Figure 1a,b). NPQ increased after 30 min of a 40 °C heat treatment (L40) compared with that under the normal condition (L25). The higher NPQ in L40 decreased to the same level as that of L25 after 30-min recovery at 25 °C in light (R25), whereas *F_v_*/*F_m_* was not affected by heat treatment in the light (Figure 1a). NPQ is subdivided into three components related to the thermal dissipation of excessive light energy (qE), state transition (qT), and photoinhibition (qI). qE and qT were major components for increasing the L40 NPQ. Thermal dissipation mediated by the xanthophyll cycle is facilitated under heat conditions to protect PSII from excess light energy [20]. An activated cyclic electron flow around PSI enhances thermal dissipation under heat stress and supports the acidification of the lumen to accelerate the xanthophyll cycle [21]. Although qT was the largest in L40, it was detected even under normal conditions in L25 and R25, coinciding with a recent report that part of the mobile LHCII in Arabidopsis is associated with PSI under all natural light conditions for plants [14]. The increasing qT in L40 suggests that heat treatment in light induces state 2 when more LHCIIs are associated with PSI.

Previous studies indicate that state transition and thylakoid membrane structure are closely related [22,23]. Transmission electron microscopy (TEM) was used to investigate the changes in the thylakoid membrane ultrastructure among various state 2 conditions in leaf chloroplasts. Thylakoid membranes in higher plants are mainly composed of two regions called appressed grana stacks and non-appressed stroma lamellae. Thylakoid membranes in L25 seedlings formed well-distinguished appressed grana stacks and stroma lamella (Figure 1c). However, many grana clearly unstacked after L40 treatment. These dissociated grana stacks seemed to be restacked within 30 min under normal conditions after heat stress, as shown in the R25 sample (Figure 1c,d). However, the height of the 10 thylakoid layers clearly differed among the three samples, *i.e.*, the L40 grana region was mostly unstacked, while the R25 grana region was more loosely stacked than the L25 grana region (Figure 1d). These results suggest that L40 is a condition in which more LHCII is associated with PSI among these three samples. Starch granules were observed in L25; however, very few starch granules were observed after heat treatment (Figure 1c,d). This result may be related to the increased activity of starch degradation. The activity of β-amylase, which is involved in starch degradation, increased under moderate heat conditions [24]. It is likely that L40 stimulates the activity of several enzymes involved in starch degradation such as β–amylase. In addition, the decrease of starch granules in L40 would be related to the inability to make starch because of an inactivated Calvin–Benson cycle at high temperatures. The production of starch granules recovered in R25 after heat stress. Plastoglobules, which are plastid-localized lipoprotein particles containing tocopherols and other lipid isoprenoid-derived metabolites, increase in number under moderate heat conditions in *A. thaliana* [25]. However, the numbers of plastoglobules in chloroplasts of wheat did not differ among the three samples (Figure 1d).

### 2.2. Phosphorylation Level of Thylakoid Proteins Increased by Heat Treatment

**Figure 2:**
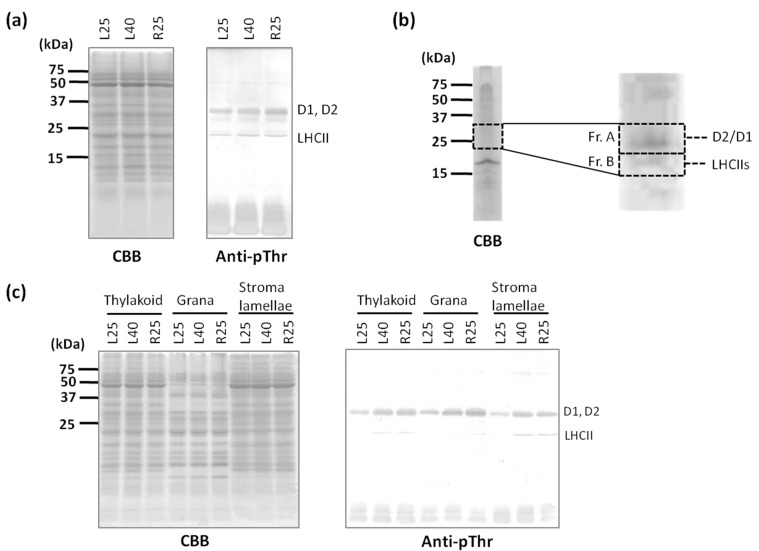
Phosphorylation level of PSII proteins increased by heat treatment. (**a**) Thylakoid membrane proteins were subjected to SDS-PAGE and subsequent immunoblotting using specific phospho-threonine antibodies; (**b**) Identification of the phosphorylated thylakoid proteins. Purified phosphorylated thylakoid proteins in L40 that were purified using the ProQ Diamond Phosphoprotein Enrichment Kit were separated by SDS-PAGE, and the bands in the dotted boxes were detected by peptide MS finger printing analysis. Mascot search results are shown in Supplementary Table S1; and (**c**) Thylakoid membrane proteins, stacked grana and unstacked stroma lamellae were subjected to SDS-PAGE and subsequent immunoblotting using specific phospho-threonine antibodies.

The increase in NPQ and qT observed in chlorophyll fluorescence analysis (Figure 1a,b) suggested that state 2 occurred in the L40 samples. During state 2 induction, PSII core proteins and LHCII are phosphorylated by thylakoid kinase STN7 and STN8, respectively [13]. To compare the phosphorylation levels of thylakoid proteins in heat-treated wheat, we performed immunobloting using anti-phosphothreonine (pThr) antibody because Thr residues are key phosphorylated amino acid residues [26]. Anti-pThr signals increased at approximately 33 and 25 kDa in the L40 samples compared with those in the L25 samples (Figure 2a). These phosphorylated thylakoid proteins were purified using the ProQ phosphorylated protein purification kit and separated by sodium dodecyl sulfate polyacrylamide gel electrophoresis (SDS-PAGE). Several bands appearing at approximately 23–35 kDa were fractionated into two fractions (Fr. A and B) and identified by peptide mass spectrometry (MS) fingerprinting analysis (Figure 2b). The sequence in fraction A was identified as D1 and D2 proteins. The sequences in Fr. B were identified as three LHCIIs, as CP26, Lhcb1, and Lhcb2 (Table S1 ).

The phosphorylation of LHCII is important for it to dissociate from PSII, which is enriched in the grana region, and is associated with PSI in stroma lamellae during state transition. To identify the location of the phosphorylated PSII core and LHCII proteins detected in the heat-treated samples, we separated thylakoid membranes into grana and stroma lamellae by digitonin fractionation and analyzed the fractions by SDS-PAGE. Heat treatment did not affect the protein profiles of any of the samples (Figure 2c, Coomassie Brilliant Blue (CBB) staining). Many phosphorylated LHCIIs were detected in both L40-treated and R25-treated stroma lamellae (Figure 2c, immunoblot). D1 and D2 proteins were detected in a much more phosphorylated state in the L40 and R25 grana. The phosphorylation of D1 and D2 proteins is believed to be correlated with the thylakoid membrane structure because the negative charge on the phosphate groups influences the surface charges on the thylakoid membranes, resulting in unstacked, appressed membrane regions caused by electrostatic repulsion [23,27]. Therefore, state transitions and the thylakoid membrane ultrastructure have been suggested to be closely related [22], and an increase in the unstacked grana region has been observed in light-induced state-2 chloroplasts. Also in heat-induced state 2 in wheat, it is possible that a relationship exists between the phosphorylation of thylakoid proteins and unstacking of thylakoid membranes.

### 2.3. Dephosphorylation of Thylakoid Proteins Was Retarded during Recovery from Heat Stress

**Figure 3:**
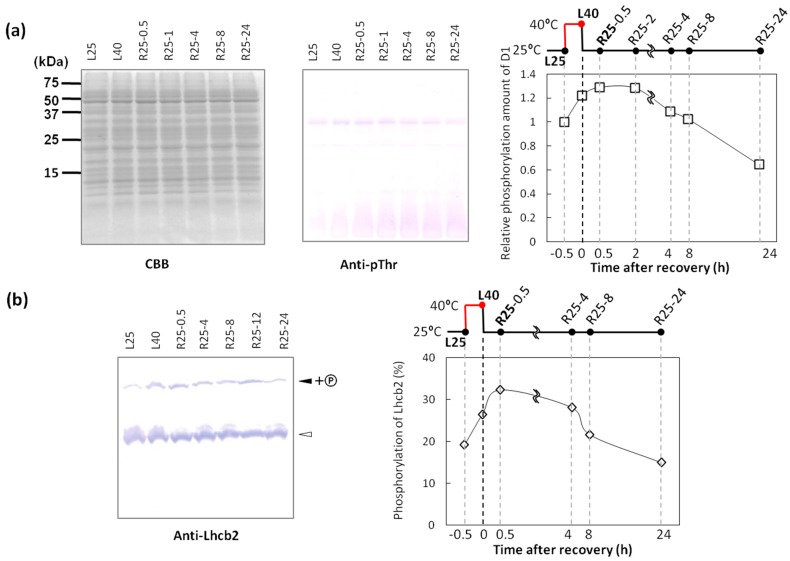
D1 and Lhcb2 were dephosphorylated during the 8 h recovery. (**a**) Proteins in the thylakoid membranes were subjected to SDS-PAGE and subsequent immunoblotting using specific phospho-threonine antibodies. Relative phosphorylation levels of D1 proteins are shown at right. *x*-axis is for the time after recovery from heat stress at 40 °C in light; (**b**) Thylakoid proteins were separated using Phos-tag™ SDS-PAGE and analyzed by immunoblotting with anti-Lhcb2 antibody. Filled arrowhead and open arrowhead shows the phosphorylated and non-phosphorylated proteins, respectively. Right panel represents the ratio of phosphorylated and non-phosphorylated Lhcb2. *x*-axis is for the time after recovery from heat stress at 40 °C in light.

The phosphorylation of thylakoid proteins (particularly PSII core proteins such as D1 and D2 proteins) in Arabidopsis under state 2 conditions plays an important role in forming unstacked thylakoid membranes [27]. However, we unexpectedly detected retarded dephosphorylation of these proteins during the 30-min recovery after heat stress (Figure 2c). It is possible that the dephosphorylation of thylakoid proteins is not important for the reconstitution of the stacked membranes. We measured the protein phosphorylation level during a prolonged recovery for 24 h to confirm the time required to dephosphorylate the PSII core and LHCII proteins (Figure 3). Phosphorylated D1/D2 and LHCII proteins were detected using anti-pThr antibody, and phosphorylated D1/D2 proteins were quantified by densitometric analysis. As shown in Figure 3a, compared with samples under normal conditions (L25), the relative phosphorylation levels of D1/D2 proteins increased under the 40 °C treatment in light (L40). The amount of D1/D2 phosphorylated proteins was the highest at 25 °C during 0.5–1 h after heat stress. The 4–8 h heat stress-recovered samples showed comparable phosphorylation levels with L25. A similar result was observed in Lhcb2 dephosphorylation analysis. Phosphorylated thylakoid proteins were separated by Phos-tag™ SDS-PAGE. In this system, phosphorylated proteins interact with Phos-tag™ co-polymerized with acrylamide in the gel, resulting in a slower migration of phosphorylated proteins than non-phosphorylated proteins [28]; thus, the ratio of phosphorylated proteins could be easily estimated. It has been reported that Lhcb2 phosphorylation plays a crucial role in inducing state 2 [29,30]; thus, we used Lhcb2 antibody to determine the Lhcb2 phosphorylation ratio in heat-stressed wheat. Densitometric analysis suggested that the shifted band occupied 19.2% of Lhcb2 before the L40 treatment, which increased to 26.4% of Lhcb2 (Figure 3b). More than 8 h were required to decrease phosphorylation to the normal level during recovery at 25 °C in light. These results suggest that the dephosphorylation of thylakoid proteins is not correlated with the reconstitution of stacked membranes during recovery at 25 °C from heat stress. This retarded dephosphorylation of thylakoid proteins could be responsible for the phosphatase of thylakoid proteins such as PPH1/TAP38 for LHCII [31,32] and PBCP for PSII core proteins [33]. The dephosphorylation process of thylakoid proteins is believed to be independent of the redox state of PQ. Therefore, reduction of the PQ pool seemed to be long-continued during recovery at 25 °C from heat stress, because the balance between the activities of these kinases and phosphatases determines the phosphorylation state of thylakoid proteins.

### 2.4. PSI–LHCII Supercomplex Increased Following Heat Stress in Light

**Figure 4:**
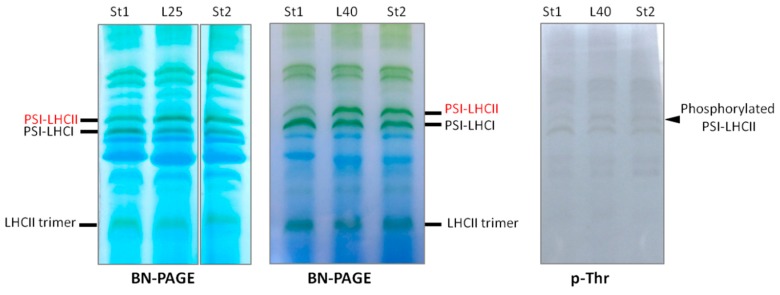
PSI–LHCII supercomplex increased by heat stress in light. Thylakoid membrane proteins were solubilized with 0.1% *n*-dodecyl-α-d-maltoside (α-DM) and 1% digitonin and subjected to Blue Native-Polyacrylamide Gel Electrophoresis (BN-PAGE) and subsequent immunoblotting using specific phospho-threonine antibodies.

Chlorophyll fluorescence and phosphoprotein analyses of wheat leaves showed that heat stress under moderate light induced state 2, which was much higher than that under growth condition in light, such as L25. In state 2, LHCII, which is bound to PSII in state 1, migrates from PSII and associates with PSI to form the PSI–LHCII supercomplex [34]. To obtain clearer evidence that L40 is in state 2, we compared the amount of the PSI–LHCII supercomplex in L40 to that in state 1 or state 2 induced by a conventional way to provoke state transition. As the conventional way to induce state transition, we used the conditions either after overnight dark adaptation at 25 °C (state 1) or after 50 min of 20 µmol photons m^−2^·s^−1^ white light treatment at 25 °C (state 2) [35]. As shown in Figure 4, thylakoid membranes were solubilized using a very mild solubilization condition (1% digitonin and 0.1% *n*-dodecyl-α-d-maltoside (α-DM)), which efficiently detects PSI–LHCII supercomplexes [35]. As a result, the amount of the PSI–LHCII supercomplex in the L40 sample was similar to that in conventionally induced-state 2 (St2). We also detected an increase in PSI-LHCII phosphorylation using p-Thr antibody, indicating that phosphorylated LHCII is bound to PSI in L40. In addition, we unexpectedly detected that the amount of the PSI–LHCII supercomplex in L25 was closer to that in St2 or L40 rather than that in conventionally induced state 1 (St1).

**Figure 5:**
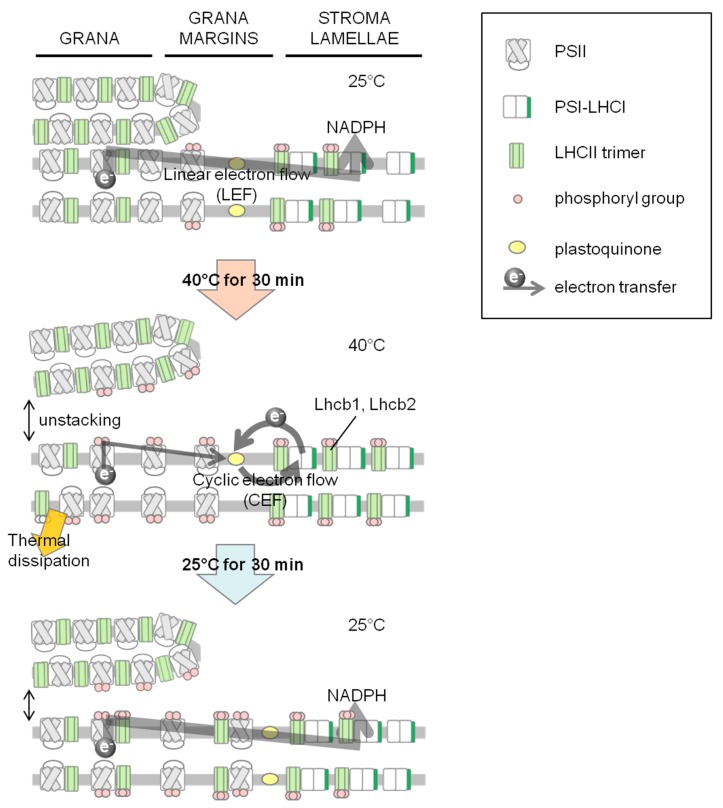
Schematic diagram of the thylakoid membrane and electron flow under different temperature conditions. **Upper** panel, at 25 °C in the light, the linear electron flow (gray arrow) is the primary pathway although most of LHCII is associated with PSI, and the thylakoid membrane is stacked; **Middle** panel, under heat-stressed condition at 40 °C in light, LHCIIs and PSII core subunits are phosphorylated. PSI, which is associated with phosphorylated LHCII trimers is preferentially excited and enhances cyclic electron flow (CEF) (gray bold arrow) to avoid over-reduction of PQ. Acidification of lumen by CEF (black arrow) induces PSII thermal dissipation (orange arrow), which restricts linear electron flow (gray thin arrow); **Lower** panel, recovery from heat stress, LHCIIs and PSII core subunits remain phosphorylated, and the thylakoid membranes are not rigidly appressed.

Figure 5 shows a hypothetical scheme of remodeling of photosystems in response to heat. Under growth conditions in light, qT and phosphorylation of thylakoid proteins were detected, and the amount of the PSI–LHCII supercomplex was similar to that in conventionally induced state 2, although linear electron flow (LEF) generates NADPH (Figure 5; upper panel). In this state, part of LHCII is phosphorylated and is associated with PSI in stroma lamellae to keep the PQ pool oxidized by smooth electron flow through PSI, and light energy would preferentially excite PSII [19]. Under high temperature conditions in light, energy distribution between the photosystems and electron transfer would presumably change. At the onset of 40 °C, the enhanced introduction of stromal reducing power into PQ may trigger additional phosphorylation of thylakoid proteins by activated protein kinase such as STN7 and STN8. The increased thylakoid protein phosphorylation level induces thermal dissipation increased by state transition, which was shown to coincide with the unstacking of thylakoid membranes (Figure 5; middle panel). In a previous study, we explained the over-reduction of the PQ pool at 40 °C in dark [19], which would breakdown oxygen-evolving complex via reactive oxygen species. The Mn cluster is a fragile complex system that can be easily destroyed by heat. Therefore, light would protect the Mn cluster by pumping protons into the lumen to induce thermal dissipation of light energy via the activation of the xanthophylls cycle. During recovery from heat stress, the phosphorylation of thylakoid proteins is maintained, and moderate unstacking of thylakoid membranes may be controlled by the balance between the ST7 and STN8 kinases and the PPH1/TAP38 and PBCP phosphatases. In this state, the energy distribution between the photosystems seems to be the same as that under normal conditions; however, it takes approximately eight hours to return to the original level of thylakoid phosphorylation under normal conditions.

## 3. Experimental Section

### 3.1. Plant Materials and Heat Treatments

Wheat (*Triticum aestivum*) seedlings were grown on Jiffy-7 (Sakata Seed, Yokohama, Japan) in a growth chamber (12 h of white light (100 µmol photons m^−2^·s^−1^), 25 °C, Biotron LH-220S, Nippon Medical & Chemical Instruments, Tokyo, Japan). Heat treatment was carried out by incubating wheat leaves at 40 °C under fluorescent white light illumination (100 µmol photons m^−2^·s^−1^). After heat treatment, plants were recovered for 30 min at 25 °C in light. Chemicals used in this research were generally of reagent grade from Wako Pure Chemical Industries (Osaka, Japan).

### 3.2. Induction of Conventional State Transition

As the conventional way to provoke state transition, we used the conditions previously described in [35]. State 1 was induced by overnight dark adaptation at 25 °C. State 2 was induced by 50 min of 20 µmol photons m^−2^·s^−1^ white light treatment at 25 °C.

### 3.3. Measurement of the Maximal Photochemical Quantum Yield of PSII and Chlorophyll Fluorescence Analysis

The maximal photochemical quantum yield of PSII was evaluated from fluorescence measurements. The ratio of variable (*F_v_* =*F_m_* − *F_o_*) to maximum fluorescence (*F_m_*) was measured using a pulse-modulated fluorometer (Junior-PAM, Heinz Walz GmbH, Effeltrich, Germany); it reflects the quantum yield of PSII [5]. Sample leaves were dark-adapted for 2 min before the measurements. Dependence of PSII quantum efficiency (ФII), electron transport rate (ETR), and NPQ of the chlorophyll fluorescence on light intensity was calculated as (*F_m_′ − F_s_*)/*F_m_′*, ФII × PFD (photon flux density) × 0.84 × 0.5, and (*F_m_* *−* *F_m_′*)/*F_m_′*, respectively [36]. Note that the fractional absorption is assumed here to be 0.84, and the ratio of PSII to PSI is assumed to be 1:1; thus, care must be exercised in making conclusions due to these two assumptions. Blue LED (wavelength of maximum emission: 450 nm) was used as the light source. A sample leaf detached from a dark-adapted plant was placed on a wet paper towel, and the temperature was controlled by a heat block. After waiting for 5 min to equilibrate temperature, chlorophyll fluorescence analysis was performed using the WinControl Program (version 3.1, Walz). Event sequence and light conditions were as follows: measuring light on (0 min, 10 µmol photons m^−2^·s^−1^), saturating pulse (0.5 min, 10,000 µmol photons m^−2^·s^−1^), actinic light on (1.5 min, 190), saturating pulse (5 min, 10,000 µmol photons m^−2^·s^−1^), actinic light off (5.5 min), and measuring light off. For the detection of qE, qT, and qI, wheat leaves were illuminated with 820 µmol photons m^−2^·s^−1^ of actinic light. NPQ can be detected as the difference between *F_m_* and the measured maximal fluorescence after a saturating light pulse during illumination of actinic light (*F_m_′*). NPQ is composed by “fast”, “middle” and “slow” quench relaxes [37]. After switching off the light, the recovery of *F_m_*′ within 2 min and 40 min was regarded as the relaxation of the qE and qT components of NPQ, respectively in wheat. qI component detected as the difference between *F_m_* and the recovery of *F_m_′* within 40 min. qE, qT and qI values were expressed the percentages of total NPQ, NPQ = qE + qT + qI. qE, qT and qI were calculated as shown in Müller, *et al.* [38].

### 3.4. Preparation of Thylakoid Membranes

Wheat leaves were homogenized with the following medium: 20 mM Tricine-NaOH, pH 7.9, 0.4 M sucrose, and 10 mM NaCl. The homogenate was filtered with two layers of gauze, and then the filtrate was centrifuged at 1000× *g* for 1 min. After the precipitate was discarded, the supernatant was centrifuged at 1500× *g* for 7 min and the resulting precipitate was used as thylakoid membranes. All procedures were performed below 4 °C in a dark room under a green light.

### 3.5. Digitonin Fractionation of Thylakoid Membranes

We used digitonin to separate stacked and unstacked thylakoids. Digitonin fractionation of thylakoid membranes and estimation of the degree of thylakoid stacking were performed as reported by Khatoon *et al.* [39]. Thylakoid membranes were suspended in 250 µg Chl/mL in a solution containing 0.2 M sorbitol, 1.5 mM K_2_HPO_4_ (pH 7.0), and 10 mM NaF. After incubation at 4 °C for 15 min, the thylakoid membranes were treated with 0.5% digitonin for 30 min on ice. Thylakoid membranes were six-fold diluted with the same solution and centrifuged at 10,000× *g* for 30 min. The amount of chlorophyll in the pellet containing stacked grana relative to that in total thylakoids represented the degree of thylakoid stacking. The supernatant was used as unstacked stroma lamellae that most PSI was recovered.

### 3.6. Protein Analysis

Protein was applied to SDS-PAGE using 12% (*w*/*v*) acrylamide gel. Phos-tag™ SDS-PAGE was performed using 8% (*w*/*v*) acrylamide gel containing 20 µM Phos-tag™ AAL-107 (Wako Pure Chemical Industries, Osaka, Japan) and 40 µM MnCl_2_. After electrophoresis, Phos-tag™ SDS-PAGE gel was gently agitated in a transfer buffer (25 mM Tris, pH 7.6, 20% methanol, 40 mM 6-amino-*n*-caproic acid, 0.02% SDS) containing 1 mM EDTA for 10 min, and subsequently resuspended in another buffer without EDTA for 10 min before electroblotting. After SDS-PAGE, proteins were electroblotted onto a polyvinylidene fluoride membrane (ATTO, Tokyo, Japan) according to the manufacturer’s instructions. Specific antibodies against plant proteins were obtained from Agrisera (Vännas, Sweden). Phospho-Thr antibody was obtained from Cell signaling technology (Beverly, MA, USA). Alkaline phosphatase-conjugated anti-rabbit or anti-mouse IgG antibody (Sigma-Aldrich, St. Louis, MO, USA) was used as a secondary antibody, and signals were visualized by bromo-chloro-indolyl phosphate and nitroblue tetrazolium. Densitometric analysis was carried out using ImageJ software (version 1.41, NIH, Bethesda, MD, USA) after scanning the blotted membrane.

### 3.7. Protein Identification by Mass Spectrometry

The thylakoid membrane fractions from wheat plants, heat-stressed at 40 °C for 30 min in the presence of light, were prepared with 20 mM Tricine-NaOH, pH 7.9, 0.4 M sucrose, and 10 mM NaCl, and then phosphorylated proteins were purified by immobilized metal affinity chromatography (IMAC) using a ProQ Phosphorylated Protein Purification Kit [40] (Invitrogen, Waltham, MA, USA). Phosphorylated protein fraction was separated by SDS-PAGE and stained with Coomassie Brilliant Blue. Next, two gel slices were excised from a sample lane in the 23–37 kDa range, followed by in-gel digestion with 10 μg/mL trypsin (Promega, Fitchburg, WI, USA) overnight at 37 °C [41]. Digested peptides were eluted with 0.1% formic acid and were subjected to LC-MS/MS analysis, which was performed on an LCMS-IT-TOF (Shimadzu, Kyoto, Japan) interfaced with a nano reverse-phase liquid chromatography system (Shimadzu, Kyoto, Japan). LC separation was performed using a Pico Frit Beta Basic C18 column (New Objective, Woburn, MA, USA) at 300 nL/min. Peptides were eluted using gradients of 5%–45% acetonitrile in 0.1% formic acid and sprayed directly into the mass spectrometer. The heated capillary temperature and electrospray voltage were set at 200 °C and 2.5 kV, respectively. MS/MS data were acquired in the data-dependent mode by LCMS solution software (Shimadzu, Kyoto, Japan) and were converted to a single text file (containing the observed precursor peptide *m*/*z*, the fragment ion *m*/*z*, and intensity values) by Mascot Distiller (Matrixscience, Boston, MA, USA). The file was analyzed using the Mascot (Matrixscience) MS/MS Ion Search to search, and the obtained peptides were assigned to the SwissProt database (SIB, Geneva, Switzerland). The search parameters were set as follows: Database, SwissProt; Taxonomy, all; Enzyme, trypsin; Variable modifications, carbamidomethyl (C), oxidation (M), propionamid (C); Peptide tol., ±0.05 Da; and MS/MS tol., ±0.05 Da. For protein identification, the criteria were as follows: (1) Mascot scores above the statistically significant threshold (*p* < 0.05) and (2) at least one top-ranked unique peptide matching the identified protein.

### 3.8. Blue Native-PAGE

Blue native-PAGE was performed as described previously in [35,42], with some minor modifications. Isolated thylakoid membranes were solubilized in 25 mM BisTris-HCl (pH 7.0), 20% glycerol, 0.25 mg/mL Pefabloc, 0.1% α-*n*-dodecyl-d-maltoside and 1% digitonin at a final chlorophyll concentration of 0.5 mg/mL. After incubation for 20 min on the ice and centrifugation at 18,000× *g* for another 20 min, the supernatants were supplemented with 1/10 volume of BN sample buffer (100 mM BisTris-HCl, pH 7.0, 5% Serva blue G, 0.5 M 6-amino-*n*-caproic acid, and 30% sucrose (*w*/*v*)), as established previously [43]. Thylakoid protein complexes were separated by NativePAGE™ Novex^®^ 3%–12% Bis-Tris Protein Gels (Invitrogen, Carlsbad, CA, USA).

### 3.9. Transmission Electron Microscopy

Leaves were trimmed into small pieces (2 × 3 mm^2^) with a razor blade; these pieces were prefixed with 2.5% glutaraldehyde (Nisshin EM, Tokyo, Japan) in 0.1 M phosphate-buffered saline (PBS) at pH 7.4 at 4 °C, overnight. Leaf pieces were then rinsed with PBS three times at intervals of 10 min and postfixed with 1% buffered osmium tetroxide (Nisshin EM, Tokyo, Japan) at room temperature for 1 h. After the samples had been rinsed briefly with distilled water, they were immediately dehydrated in ethanol series (50%, 70%, 90%, and 100%). They were then immersed in an intermediate solvent (propylene oxide; Nisshin EM, Tokyo, Japan) for 10 min and in a mixture (1:1, *v*/*v*) of propylene oxide and Spurr’s resin [44] (Polysciences, Warrington, PA, USA) for 6 h at room temperature and then placed in pure Spurr’s resin at 4 °C for 3 days, and then embedded in plain embedding plates and polymerized at 70 °C for 24 h.

Blocks of leaves were cut with a Porter-Blum MT-1 ultramicrotome (Ivan Sorvall, Norwalk, CT, USA) and a diamond knife (Diatome, Bienne, Switzerland). Ultrathin sections about 80 nm were prepared; these were stained with 4% aqueous UA for 10 min at room temperature and Sato’s lead for 10 min [45]. Every staining step was succeeded by a step of washing with water three times for 3 min. The stained as well as the unstained sections were observed under a JEM-1400 electron microscope (JEOL, Akishima, Japan) at an accelerating voltage of 75 kV.

## 4. Conclusions

Photosystems of higher plants alleviate heat-induced damage in the presence of light under moderate stressed conditions. To obtain a better understanding of heat tolerance of the photosystem, we focused on the regulating photochemical energy transfer in heat-treated wheat at 40 °C with light. Structural changes of thylakoid membrane accompanied by the phosphorylation of thylakoid proteins such as D1 and D2 proteins and the LHCII proteins Lhcb1 and Lhcb2 would assist the remodeling of photosystems and regulation of energy distribution by transition toward state 2 probably contributes to plastoquione oxidation; thus, light-driven electrons flowing through PSI play a protective role against PSII damage under heat stress. In this research, state 1 to state 2 transition was observed under high temperature in light. Further research is expected to elucidate the detailed mechanism of heat stress-induced state transition e.g., distinguish between light intensity-induced and heat stress-induced state transition. 

## Supplementary Materials

Supplementary table can be found at http://www.mdpi.com/1422-0067/15/12/23042/s1.
